# Supplementary material for: Pyruvate Dehydrogenase Kinase 1 inhibition mediated oxidative phosphorylation enhancement in cartilage promotes osteoarthritis progression
Source: BMC Musculoskelet Disord. 2023 Jul 20;24:597. doi: 10.1186/s12891-023-06585-6 (PMC10357736; doi:10.1186/s12891-023-06585-6)
Supplement: Supplementary file 1 — Additional file 1. [file 12891_2023_6585_MOESM1_ESM.zip › MF.Gsea.1653627316242/GOMF_NUCLEAR_LOCALIZATION_SEQUENCE_BINDING.html]

Details for gene set GOMF\_NUCLEAR\_LOCALIZATION\_SEQUENCE\_BINDING[GSEA]

|  || Dataset | 55457\_collapsed\_to\_symbols.cls.cls #OA\_versus\_normal.cls.cls #OA\_versus\_normal\_repos |
| Phenotype | cls.cls#OA\_versus\_normal\_repos |
| Upregulated in class | normal |
| GeneSet | GOMF\_NUCLEAR\_LOCALIZATION\_SEQUENCE\_BINDING |
| Enrichment Score (ES) | -0.56707203 |
| Normalized Enrichment Score (NES) | -1.7928983 |
| Nominal p-value | 0.005172414 |
| FDR q-value | 0.076415665 |
| FWER p-Value | 0.579 |
Table: GSEA Results Summary

  

Fig 1: Enrichment plot: GOMF\_NUCLEAR\_LOCALIZATION\_SEQUENCE\_BINDING      
 Profile of the Running ES Score & Positions of GeneSet Members on the Rank Ordered List

  

| SYMBOL | TITLE | RANK IN GENE LIST | RANK METRIC SCORE | RUNNING ES | CORE ENRICHMENT || 1 | POM121L2 | POM121 transmembrane nucleoporin like 2 [Source:HGNC Symbol;Acc:HGNC:13973] | 2841 | 0.266 | -0.1908 | No |
| 2 | RANBP6 | RAN binding protein 6 [Source:HGNC Symbol;Acc:HGNC:9851] | 3179 | 0.233 | -0.1909 | No |
| 3 | IPO5 | importin 5 [Source:HGNC Symbol;Acc:HGNC:6402] | 6949 | -0.076 | -0.4751 | No |
| 4 | KPNA5 | karyopherin subunit alpha 5 [Source:HGNC Symbol;Acc:HGNC:6398] | 7062 | -0.085 | -0.4744 | No |
| 5 | NPAP1 | nuclear pore associated protein 1 [Source:HGNC Symbol;Acc:HGNC:1190] | 7737 | -0.136 | -0.5114 | No |
| 6 | KPNA1 | karyopherin subunit alpha 1 [Source:HGNC Symbol;Acc:HGNC:6394] | 8260 | -0.176 | -0.5323 | Yes |
| 7 | KPNB1 | karyopherin subunit beta 1 [Source:HGNC Symbol;Acc:HGNC:6400] | 8285 | -0.177 | -0.5143 | Yes |
| 8 | NUP214 | nucleoporin 214 [Source:HGNC Symbol;Acc:HGNC:8064] | 8301 | -0.178 | -0.4955 | Yes |
| 9 | CABP1 | calcium binding protein 1 [Source:HGNC Symbol;Acc:HGNC:1384] | 9089 | -0.239 | -0.5299 | Yes |
| 10 | KPNA6 | karyopherin subunit alpha 6 [Source:HGNC Symbol;Acc:HGNC:6399] | 9221 | -0.250 | -0.5121 | Yes |
| 11 | KPNA2 | karyopherin subunit alpha 2 [Source:HGNC Symbol;Acc:HGNC:6395] | 9753 | -0.297 | -0.5201 | Yes |
| 12 | KPNA3 | karyopherin subunit alpha 3 [Source:HGNC Symbol;Acc:HGNC:6396] | 10359 | -0.357 | -0.5271 | Yes |
| 13 | BRAP | BRCA1 associated protein [Source:HGNC Symbol;Acc:HGNC:1099] | 10594 | -0.383 | -0.5024 | Yes |
| 14 | NUP98 | nucleoporin 98 and 96 precursor [Source:HGNC Symbol;Acc:HGNC:8068] | 10875 | -0.413 | -0.4779 | Yes |
| 15 | NOLC1 | nucleolar and coiled-body phosphoprotein 1 [Source:HGNC Symbol;Acc:HGNC:15608] | 10942 | -0.423 | -0.4356 | Yes |
| 16 | IPO4 | importin 4 [Source:HGNC Symbol;Acc:HGNC:19426] | 11119 | -0.445 | -0.3994 | Yes |
| 17 | NUP58 | nucleoporin 58 [Source:HGNC Symbol;Acc:HGNC:20261] | 11132 | -0.447 | -0.3503 | Yes |
| 18 | NUP153 | nucleoporin 153 [Source:HGNC Symbol;Acc:HGNC:8062] | 11307 | -0.472 | -0.3110 | Yes |
| 19 | KPNA4 | karyopherin subunit alpha 4 [Source:HGNC Symbol;Acc:HGNC:6397] | 11395 | -0.486 | -0.2633 | Yes |
| 20 | POM121 | POM121 transmembrane nucleoporin [Source:HGNC Symbol;Acc:HGNC:19702] | 12146 | -0.631 | -0.2510 | Yes |
| 21 | TNPO2 | transportin 2 [Source:HGNC Symbol;Acc:HGNC:19998] | 12227 | -0.652 | -0.1841 | Yes |
| 22 | NFKBIA | NFKB inhibitor alpha [Source:HGNC Symbol;Acc:HGNC:7797] | 12817 | -1.001 | -0.1177 | Yes |
| 23 | TNPO1 | transportin 1 [Source:HGNC Symbol;Acc:HGNC:6401] | 12864 | -1.108 | 0.0028 | Yes |
Table: GSEA details [plain text format]

  

Fig 2: GOMF\_NUCLEAR\_LOCALIZATION\_SEQUENCE\_BINDING      
 Blue-Pink O' Gram in the Space of the Analyzed GeneSet

  

Fig 3: GOMF\_NUCLEAR\_LOCALIZATION\_SEQUENCE\_BINDING: Random ES distribution      
 Gene set null distribution of ES for **GOMF\_NUCLEAR\_LOCALIZATION\_SEQUENCE\_BINDING**
